# Altered *ARID1A* expression in colorectal cancer

**DOI:** 10.1186/s12885-020-6706-x

**Published:** 2020-04-25

**Authors:** Mehran Erfani, Seyed Vahid Hosseini, Maral Mokhtari, Mozhdeh Zamani, Kamran Tahmasebi, Mahvash Alizadeh Naini, Alireza Taghavi, John M. Carethers, Minoru Koi, Hassan Brim, Pooneh Mokarram, Hassan Ashktorab

**Affiliations:** 1 Autophagy Research Center and Department of Biochemistry, Shiraz University of Medical Sciences, Shiraz, Iran; 2 Colorectal Research Center, School of Medicine, Shiraz University of Medical Sciences, Shiraz, Iran; 3 Department of Pathology, School of Medicine, Shiraz University of Medical Sciences, Shiraz, Iran; 4 Department of Internal Medicine, Gastroenterology division, School of Medicine, Shiraz University of Medical Sciences, Shiraz, Iran; 5 Departments of Internal Medicine and Human Genetics, and Rogel Cancer Center, University of Michigan, Ann Arbor, MI 48109-5368 USA; 6 Cancer Center and Department of Medicine, Howard University, College of Medicine, 2041 Georgia Avenue, N.W., Washington, D.C. 20060 USA; 7 Autophagy Research Center, Shiraz University of Medical Sciences, Shiraz, Iran

**Keywords:** ARID1A, Methylation, Colorectal cancer, Immunohistochemistry

## Abstract

**Background:**

ARID1A has been described as a tumor suppressor gene, participating in chromatin re-modeling, epithelial-mesenchymal-transition and many other cellular and molecular processes. It has been cited as a contribute in tumorigenesis. The role of ARID1A in CRC is not yet defined.

**Aim:**

To investigate the role of *ARID1A* methylation and CNV in its expression in CRC cell lines and to examine the relationship between ARID1A status with survival and clinicopathologic characteristics in patients with CRC.

**Methods:**

We used RT-PCR to determine both CNV and expression of ARID1A from six CRC cell lines. We used MSP to evaluate methylation of *ARID1A*. IHC was used to assess ARID1A protein expression. We also evaluated MSI and EMAST status in 18 paired CRC and adjacent normal tissues. 5AzadC was used to assess effect of DNA demethylation on ARID1A expression. Statistical analysis was performed to establish correlations between ARID1A expression and other parameters.

**Results:**

Among the 18 CRC tumors studied, 7 (38.8%) and 5 tumors (27.7%) showed no or low ARID1A expression, respectively. We observed no significant difference in ARID1A expression for overall patient survival, and no difference between clinicopathological parameters including MSI and EMAST. However, lymphatic invasion was more pronounced in the low/no ARID1A expression group when compared to moderate and high expression group (33% VS. 16.6% respectively. *ARID1A* promoter methylation was observed in 4/6 (66%) cell lines and correlated with *ARID1A* mRNA expression level ranging from very low in SW48, to more pronounced in HCT116 and HT-29/219. Treatment with the methyltransferase inhibitor 5-Azacytidine (5-aza) resulted in a 25.4-fold and 6.1-fold increase in *ARID1*A mRNA expression in SW48 and SW742 cells, respectively, while there was no change in SW480 and LS180 cells. No *ARID1A* CNV was observed in the CRC cell lines.

**Conclusion:**

ARID1A expression is downregulated in CRC tissues which correlates with it being a tumor suppressor protein. This finding confirms ARID1A loss of expression in CRC development. Our in-vitro results suggest high methylation status associates with reduced ARID1A expression and contributes to CRC tumorigenesis. However, there was no significant association between ARID1A loss of expression and clinicopathological characteristics. Future in-vivo analysis is warranted to further establish ARID1A role in colorectal neoplastic transformation.

## Introduction

Colorectal cancer (CRC) is rapidly increasing in prevalence among several regions of the world, such as Asia [1, 2]. CRC is the second and the third major reason for cancer-related death among men and women, respectively [3, 4]. The molecular pathogenesis of CRC is driven by successive acquisition of epigenetic and genetic changes, which associate with silencing of tumor suppressor genes and activation of proto-oncogenes [5]. The low efficiency of conventional therapies to increase longevity in CRC patients, calls for new targeted treatment. Characterization of genes related to the progression and development of colon cancer showed that their clinical implication is crucial for diagnosis and efficient therapy.

*ARID1A* encodes the AT-rich interactive domain-containing protein 1A, a representative of the DNA-binding protein family and principal subunit of the SWI–SNF complex (switch/sucrose non-fermentable). *ARID1A* is frequently deleted in multiple human tumors. It is located on chromosome 1p36.11, a region that is commonly deleted in various cancer types and suspected to contain tumor suppressor genes [6–10]. For example, deletion of 1p36 region, harboring *ARID1A*, was observed in more than half pancreatic carcinomas [11, 12]. SWI–SNF is a highly conserved chromatin rearranging complex, applying adenosine triphosphate (ATP)-dependent helicase functions to facilitate the access of transcriptional repressors and activators to DNA [13, 14]. Therefore, the protein interferes with regulation processes, including development, differentiation and DNA repair [15]. ARID1A protein is typically deficient in oncogenic tissues, and therefore is suspected to possess the tumor suppressor activity of the complex [16, 17]. Also, ARID1A plays a critical role in the FAS-mediated apoptosis [18]. The interplay between gene and promoter is associated with the inhibition of cell-cycle-specific genes [19]. *ARID1A* knock-out cells do not enter cell cycle arrest [20]. The ARID1A involvement in cell-cycle arrest shows that it significantly aids tumor repression, through the SWI/SNF complexes [17]. A wide range of cancer-gene mutations are detected by NGS and loss of function mutations in the *ARID1A* is detected repeatedly and frequently in various cancer types [13, 21]. A recent study revealed that *ARID1A* knockdown in renal cells led to epithelial-mesenchymal-transition, highlighting its role in cell differentiation and tissue homeostasis [22].

Although ARID1A expression loss has been described chiefly in gynecological cancers, it is reported among other tumor types, such as from gastrointestinal tract tumors [23–25]. In gastric and gynecological cancers, *ARID1A* mutation or loss of ARID1A protein expression strongly correlates with microsatellite instability, and is inversely correlated with alterations in TP53 [23, 26]. Recently, there has been a growing interest in clinical significance of ARID1A low expression in gastrointestinal oncogenic conditions, particularly tumors manifesting DNA mismatch repair (MMR) deficiency [27].

Molecular mechanisms related to low ARID1A expression seem to be distinct amongst different cancerous tumors. For instance, *ARID1A* copy number loss is the major cause of low ARID1A expression in pancreatic cancer (47%) [28]. Prior studies indicate that copy number loss exists in 13–35% of breast cancers [17]. Mutation can also affect the expression in ovarian clear cell carcinoma, with *ARID1A* mutation (with 50% mutation rate) as the major cause of loss of expression. Moreover, among breast cancers, promoter hypermethylation and histone modification are the main reasons for loss of ARID1A expression [17].

Studies on ARID1A expression in CRC is limited. A relatively high mutation rate of *ARID1A* was reported in the colorectal cancer (10–40%) [13, 29–31], but it is not apparent whether DNA hypermethylation, and/or copy number variation (CNV) also are contributory in alteration of ARID1A expression. To explore the major molecular mechanisms of ARID1A expression loss in CRC, we aimed to study *ARID1A* methylation, expression and CNV in clinical samples and CRC cell lines. We also determine if treatment of these cell lines with 5-aza affect the expression of ARID1A. In addition, we examined possible correlations between ARID1A expression loss and various clinicopathological parameters in CRC tissues.

## Materials and methods

### Patient

Eighteen paraffin-embedded patient-derived paired CRC and adjacent nontumorous tissue samples were collected from the archives of the department of pathology of Faghihi Hospital of Shiraz University of Medical Sciences. All patients underwent primary tumors resection between 2016 and 2017. None of the patients had preoperative radiotherapy or preoperative chemotherapy. Tumor staging was determined according to AJCC TNM system. All tumors were histologically classified based on World Health Organization criteria. Clinical, pathologic and follow-up information of patients were obtained from hospital medical records. Overall survival (OS) was defined as the time interval (in months) from surgery to the time of death from any cause or to end of follow-up if the patient was alive (censored). Twelve separate cancer-matched normal pairs were from Howard University Hospital and used for exome sequencing [32]. The IRB committee of the Medical University of Shiraz and Howard University approved this study and the archival tissue were obtained, de-identified prior to receipt and there is no access to the identifiers (IRB-06-MED-39).

### Immunohistochemistry and scoring

Immunohistochemistry was performed on 4-μm thick paraffin-embedded tissue sections from patients with colorectal cancer using a rabbit anti-human ARID1A antibody (HPA005456, Sigma, USA) at a dilution of 1:200. Briefly, sections were deparaffinized using xylene and rehydrated in a descending series of alcohol dilutions. Activity of endogenous peroxidase was inhibited with 3% hydrogen peroxide in methanol for 5 min. After, the sections were heated for 15 min at 120 °C in 10 mM citrate buffer for antigen retrieval. Sections were then blocked with goat serum 1:100 in PBS for 20 min and subsequently incubated with primary antibodies for 30 min at room temperature. After washing, immunohistochemical staining was performed using Master Polymer Plus Detection System kit (Incl. DAB Chromogen) according to the manufacturer’s instructions. Sections were then counterstained with hematoxylin, dehydrated by ethanol and mounted.

Immunostained sections were reviewed and scored by an expert pathologist who was blinded to patient’s clinicopathological and molecular information, according to the percentage of the stained cells and the intensity of staining. The percentage of positively stained cells was scored as: ≤ 10% = 0, 11–25% = 1, 26–50% = 2, 51–75% = 3, and > 75% = 4. Staining results were considered positive for tumor tissues with nuclear staining. Staining intensity was scored as negative = 0, weak = 1, moderate = 2, and strong = 3. The final score of each section equals the percentage score × intensity score. Based on the overall score, the immunostained sections were further divided into two groups: The group of ARID1A-negative (overall score = 0) and the group of ARID1A-positive (overall score ≥ 1). The group of ARID1A-positive was further divided into three subgroups: low ARID1A expression group (overall score = 1–4), moderate ARID1A expression group (overall score = 5–8) and high ARID1A expression group (overall score = 9–12).

### MSI and EMAST analysis

MSI and EMAST analysis using DNA extracted from 18 paraffin-embedded patient-derived paired CRC and adjacent nontumorous tissue samples were performed as described previously [33, 34]. Briefly, MSI and EMAST status were analyzed using the standard panel for MSI detection in CRC samples (NR21, NR22, NR27, BAT25 and BAT26) and five EMAST markers (D9S242, D8S321, D20S82, D20S85 and MYCL1), respectively [35]. Microsatellite loci were amplified via PCR with previously described primers [33, 34]. Samples were classified as microsatellite instability high (MSI-H), microsatellite instability low (MSI-L), or microsatellite stable (MSS) based on the number of markers displaying instability and corresponding to ≥2 markers, one, or none, respectively. Samples were labeled EMAST-positive when at least two of the 5 the analyzed non-mononucleotide markers were unstable.

### Cell cultures

In this study, six human CRC cell lines (HCT116, HT-29/219, SW48, SW742, SW480 and LS180) were purchased from the National Cell Bank of Iran (Pasteur Institute, Tehran, Iran). cells were cultured in RPMI 1640 (HCT116, SW48, SW742, HT29/219 and SW480) or DMEM (LS180), containing 10% fetal bovine serum, 1% penicillin, 2 mM glutamine, and 1% streptomycin, in a humidified CO2 (5%) incubator at 37 °C.

### 5-Aza-C treatment

Cells were cultured in T-25 flasks, after 24 h, cells were covered for 72 h with 2.5 μM 5-Azacytidine (5-aza) (Sigma), a methyltransferase inhibitor. The culture media were replaced every 24 h with fresh media, containing 5-aza. Stock solutions of 5-aza were prepared fresh every day, sterilized by filtration, and kept at 0 °C, until further use. For drug treatment, 5-aza was dissolved in acetic acid: water (1:1 v/v). Then, the stock solution (100 mM) was diluted, up to a final concentration of 3 μM in PBS and the cells treated with 5-aza for 3 day.

### Extraction of DNA and methylation analysis

Genomic DNA was extracted from cells as follows: Cells were collected and initially lysed in 1.0 ml DNA lysis buffer (0.5% Triton X-100, 10 mM EDTA, 50 mM HEPES at pH 8), supplemented with 100 μl 1% SDS and 200 μg/mL proteinase K (Sigma-Aldrich). Following the addition of RNAse A (100 μg/mL), samples were incubated at 65 °C for 1 h. Subsequently, an equal volume of phenol:chloroform: isoamyl alcohol (25:24:1 v/v), was added to the cell lysate, shaken vigorously, and then spun at 12000 rpm for 10 min. Afterward, the supernatant was collected, and the isolated DNA was precipitated by adding 2 volumes of 100% ethanol and sodium acetate, at a concentration of 300 μM, and kept at − 20 °C for 24 h. The DNA was next dissolved in 50 μl of DNAse-free water and quantified with a NanoDrop ND-1000 spectrophotometer (Thermo Fisher Scientific, USA). The methylation status of CpG islands in the *ARID1A* promoter region was analyzed, using MS-PCR as previously described [36]. Briefly, the method detects methylation of bases in a bisulfite reaction, and subsequently detects them by polymerase chain reaction (PCR), using specific primers designed for bisulfite-converted methylated and unmethylated sequences.

**Fig. 1:**
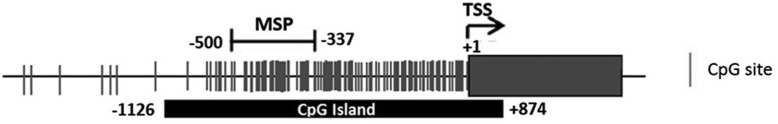
Schematic structure of the *ARID1A* CpG island, and the MSP region indicated. Each vertical line represents one CpG site. The transcription start site (TSS) is defined as position + 1, and the rest of the sequence is numbered relative to the TSS

The primer sequences used for the detection of *ARID1A* promoter region included: methylated, forward: 5′-GGCGTAGGTTTTAGAGATGC-3′, and reverse: 5′- AAACGAACTCGCTCCCTT-3′; unmethylated, forward: 5′-GGTGTAGGTTTTAGAGATGT-3′; and reverse: 5′ - AAACAAACTCACTCCCTT- 3′. The MS-PCR primers were designed using a methylation specific software (www.mspprimers.org). PCR reactions were performed in triplicates, using the following conditions: 95 °C for 10 min, and 35 cycles of 95 °C for 40 s, 59 °C (unmethylated DNA) or 53 °C (methylated DNA) for 40 s, and 72 °C for 30 s, followed by an extension at 72 °C for 10 min. A band size of 163 bp was observed in the *ARID1A* methylation set (Fig. 1). Distance of the 5′ nucleotide of the sense primers of methylated and unmethylated from the transcription start site (TSS) of the *ARID1A* was − 500 and − 498, respectively. The PCR amplicons were electrophoresed on 2% agarose gel, dyed with GelRed (Biotium, Belgium) and visualized, using the UV illumination system. Genomic DNA extracted from peripheral blood leukocytes, served as an unmethylated control sample. Human methylated DNA from Zymo Research (ZYMO Research, Freiburg, Germany), was used as methylated positive control. Distilled water was used as a blank control for the PCR reaction.

### RNA extraction and reverse transcription

Total RNA was extracted from sub-confluent cell cultures, using the Tripure RNA isolation kit (Roche Applied Science, Germany), based on the manufacturer’s guidelines. The isolated RNA was quantified by a NanoDrop ND-1000 spectrophotometer (NanoDrop Technologies). Furthermore, RNA integrity was examined by the presence of the 28S, 18S rRNA bands on 1.5% agarose gel. The cDNA was synthesized, with DNase-treated RNA, 2 μg of RNase-free, random primers or random hexamers, using the RevertAid First Strand cDNA Synthesis Kit (Fermentas), in 20 μl volumes reaction, based on the manufacturer’s instructions.

### Analysis of gene expression

Real-time quantitative PCR (qPCR) was carried out, using ABI 7500 Sequence Detection System (Applied Biosystems, USA) and the following primers for the *ARID1A* mRNA analysis: forward, 5′-CAGTACCTGCCTCGCACATA-3′; reverse, 5′ GCCAGGAGACCAGACTTGAG- 3′. Additionally, GAPDH was used as the internal housekeeping gene with the following primers: forward, 5′- CGACCACTTTGTCAAGCTCA- 3′, reverse, 5′- AGGGGTCTACATGGCAACTG-3′. The PCR reaction mixture contained 1 μl cDNA of each sample, 1 μl of forward and reverse primers (0.2 μmol L-1 final concentration) and 12.5 μl SYBR Green master mix (ABI, UK) in a total final volume of 25 μl. PCR was carried out using the following conditions: a pre-cycling thermal activation at 95 °C for 15 min, and 40 cycles of denaturation at 95 °C for 15 s, annealing at 60 °C for 30 s, extension at 72 °C for 30 s, and a final extension of 72 °C for 10 min, followed by a melt curve (58–95 °C) analysis. Experiments were performed in duplicate. Furthermore, products specificity was confirmed by electrophoresis on 2% agarose gel. The relative expression was calculated, using the 2^-ΔΔCT^ method and normalized to the GAPDH expression.

### Copy number variation analysis of *ARID1A*

Real time quantitative PCR (qPCR) was carried out to reveal the relative changes in *ARID1A* copy numbers among six CRC cell lines and a normal colon tissue sample. Ten nanograms of genomic DNA from each cell line and normal colon tissue was used as template to carry out the real time PCR reaction, using specific primer for *ARID1A*, and RNase P (two copies in a diploid genome) as a reference gene. Primers used for the RNase P are as follows: Forward 5′-AGATTTGGACCTGCGAGCG-3′ and reverse 5′- GAGCGGCTGTCTCCACAAGT-3′, giving an amplicon size of 65 bp. Primers used for *ARID1A* are forward, 5′- GGATGGCACTTTGAGAAC − 3′ and reverse 5′- GAAAGATGAACAGGAGGA − 3′, giving a fragment length of 166 bp. Amplification of the product was carried out with the following conditions: a pre-cycling thermal activation at 95 °C for 15 min, accompanied by 40 cycles of 95 °C for 15 s, 60 °C for 30 s, 72 °C for 30 s, and a final extension of 72 °C for 10 min. Experiments were performed in triplicates.

For all experiments, cycle threshold (Ct) values are extracted, with 7500 software (Applied Biosystems). Relative CNVs were computed, using the 2^-ΔΔCT^ method, which were normalized to a calibrator sample (normal sample) to have normal diploid genomes) and a reference gene (RNase P). In comparison to the normal sample, a two-fold decrease or increase in the copy number of *ARID1A* in the CRC cell lines was referred to as a deletion or amplification, respectively.

### Statistical analysis

Data analysis was performed, using SPSS software (Version 23). Data are presented as mean ± SD. The association between ARID1A expression and the clinicopathological characteristics were evaluated by Pearson χ2 test and Fisher’s exact test based on the type of data. The Kaplan-Meier method was used for survival rates estimation. Other experiments were analyzed, using the one-way ANOVA, accompanied by Tukey’s multiple comparison tests. Differences with a *P* value ≤0.05 were statistically significant.

## Results

### Differential expression of ARID1A in CRC cell lines

**Fig. 2:**
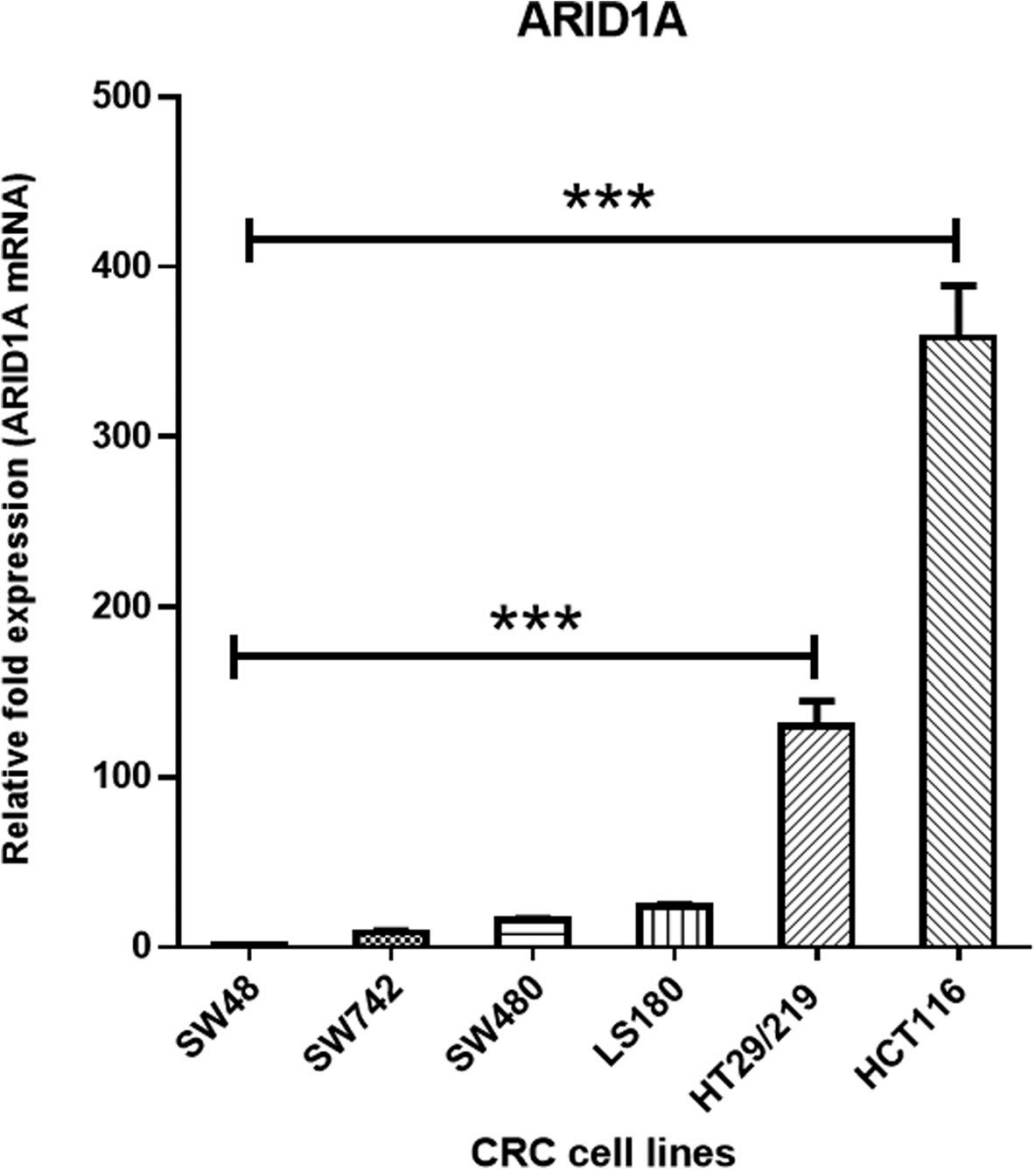
*ARID1A* relative mRNA expression in SW742, LS180, HCT116, HT29/219, SW480 and SW48 cell lines was determined by Real-Time RT-PCR. Each gene’s expression values was normalized to the GAPDH mRNA. SW48 cell line was used as a reference, with an expression level set to 1.0, and expressions in all other cell lines were presented as an n-fold difference, compared to the SW48. Mean ± SD of three experiments is reported (*p* < 0.05)

**Fig. 3:**
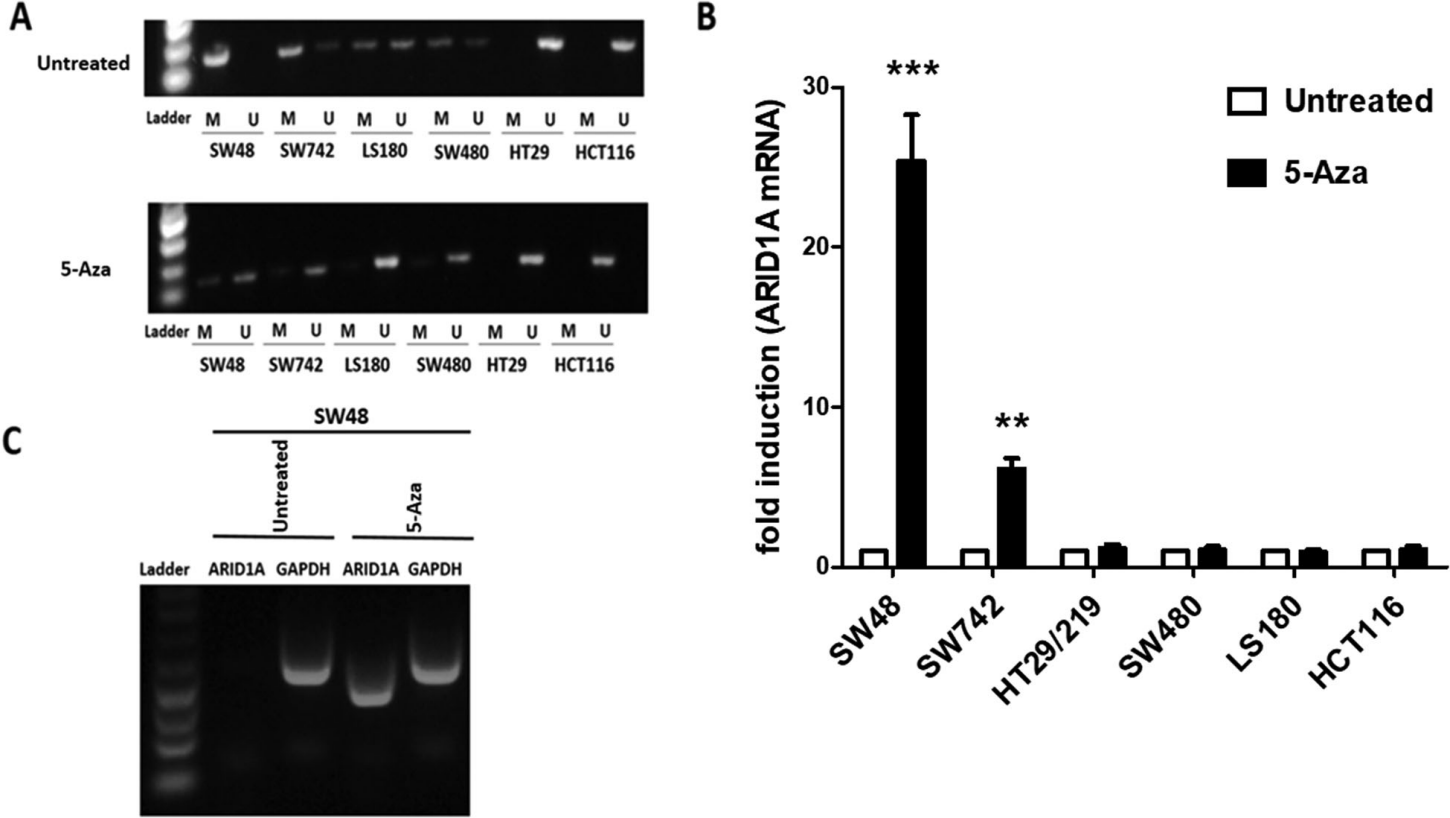
Methylation and mRNA expression status of *ARID1A* before and after treatment with 5-aza in CRC cell lines. **a** Methylation status of the *ARID1A* promoter was examined by MSP in CRC cell lines. The methyltransferase inhibitor (5-Aza) induced demethylation in CRC cell lines by MSP, U: unmethylated, M: methylated. **b** Treatment with 5-Aza alters the *ARID1A* mRNA expression in a cell line-dependent manner. Relative *ARID1A* mRNA expression before and after 5-Aza treatment were examined by Real-Time RT-PCR. *ARID1A* mRNA expression was normalized to GAPDH mRNA. Demethylation treatment restored *ARID1A* mRNA expression in SW48, SW742, LS180, HT29 cell line by RT-PCR. The data were presented as average fold-changes ± SD. **c***ARID1A* mRNA expression is silenced in SW48 cell line

The *ARID1A* mRNA expression was examined in six colorectal cancer cell lines, SW48, SW742, HT29/219, HCT116, LS180, and SW480 (Fig. 2). *ARID1A* mRNA average levels (normalized to GAPDH mRNA), showed that SW48 cells expressed at the lowest levels. As shown in Figs. 2 and 3c, *ARID1A* mRNA expression was not observed in SW48 cells and therefore were used as baseline/reference for *ARID1A* expression levels (set at 1.0). The average expression level of the *ARID1A* mRNA in HCT116 cells was significantly higher (364 fold; *P* < 0.05) in comparison with the other cell lines. Quantitation of the *ARID1A* mRNA revealed that HT29/219, LS180, SW480 and SW742 cells, expressed approximately, 136.8, 24.3, 15.5 and 8.6- fold more than the SW48 cells, respectively. We observed a spectrum of high-level expression (in HCT116) to virtually no expression (in SW48) for *ARID1A* mRNA (Fig. 2).

### *ARID1A* MSP analysis shows different methylation level in CRC cell lines

Since aberrant methylation plays a key role in regulating *ARID1A* expression in several cancers [17], we examined the methylation status of the *ARID1A* promoter in the six CRC cell lines by methylation specific PCR (MSP). MSP showed that SW48 cells were fully methylated (Fig. 3a); completely downregulating *ARID1A* mRNA expression. No *ARID1A* promoter methylation was detected in the HCT116 and HT29 cell lines (Fig. 3a). SW742, SW480 and LS180 cell lines displayed both methylated and unmethylated alleles (Fig. 3a). Coincident with HCT116 cells expressing the highest level of ARID1A (Fig. 2), there was no promoter methylation; SW48 cell matched *ARID1A* transcript downregulation with full methylation of the *ARID1A* promoter.

### Expression analysis of ARID1A after treatment with 5-Aza-C

To determine if aberrant promoter methylation is the process affecting *ARID1A* mRNA expression, cells were treated with the DNA methyltransferase inhibitor 5-aza, and *ARID1A* expression was examined by real-time RT-PCR. SW48 cells that has full methylation dramatically restored *ARID1A* expression (25.4 fold) after 5-aza treatment (Fig. 3b and c) while HCT116 cells that had no *ARID1A* promoter methylation, did not show *ARID1A* expression changes (Fig. 3b). SW742 cells that show partial methylation, increased *ARID1A* expression by 6.1-fold with 5-aza treatment (Fig. 3a, b). Treating SW48, SW742, LS180 and SW480 cells with 5-aza resulted in an increase in the unmethylated PCR product due to reduction of *ARID1A* methylation but no overall mRNA expression (Fig. 3a and b).

### Colorectal cancer cell lines show unaltered *ARID1A* copy numbers

During carcinogenesis, the gain or loss of *ARID1A* copy numbers could be the basis for variations in gene expression. In order to examine whether differences in basal *ARID1A* expression, was the consequence of *ARID1A* copy number variations, we employed real time PCR. Colorectal cancer cell lines, SW48, SW742, HT29/219, HCT116, LS180 and SW480 cells were analyzed with a normal DNA sample of colon tissue. The *ARID1A* copy numbers were normalized and calculated relative to the endogenous reference gene RNase P, which is shown to be available in two copies in a diploid genome.

**Fig. 4:**
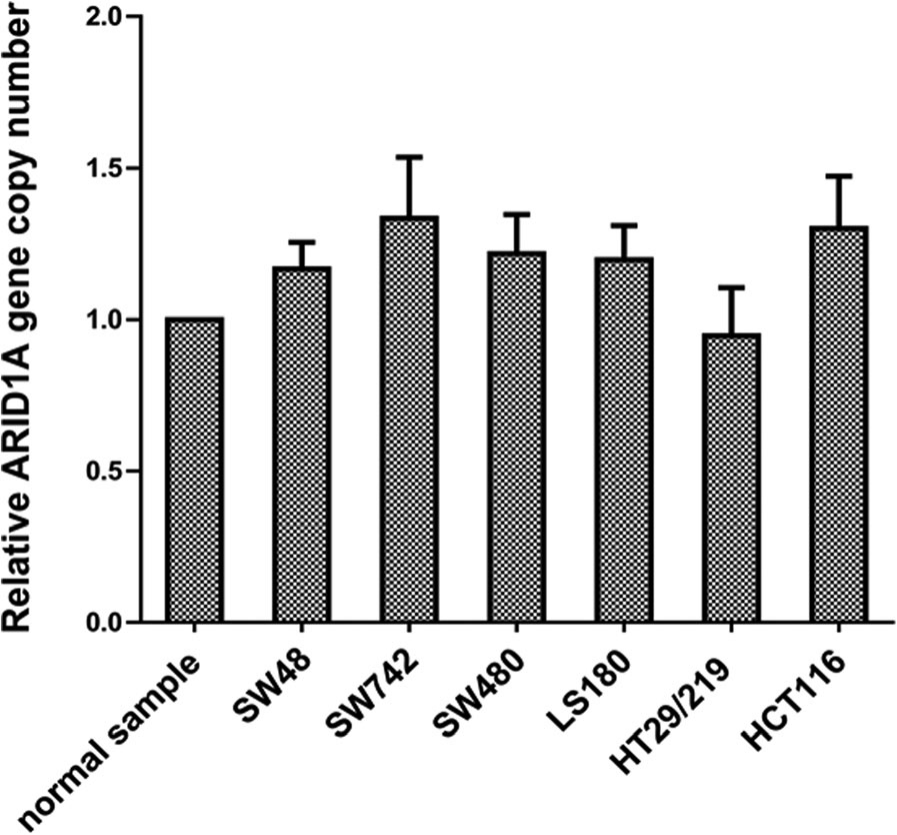
Analysis of *ARID1A* copy number variation in the CRC cell lines. CRC cell lines: LS180, SW742, HCT116, HT29/219, SW480 and SW48 were analyzed relative to a DNA sample of a normal colon tissue, regarding the copy number variation (CNV) of *ARID1A*, using real-time PCR. In the CRC cell lines, *ARID1A* copy numbers were comparable with the control

The comparison between *ARID1A* copy number of CRC cell lines related to control revealed that CRC cell lines had no significant difference in *ARID1A* copy number (Fig. 4). These data suggest that the CNV is unlikely to be involved in the differences in ARID1A expression in the studied CRC cell lines.

### ARID1A expression was frequently reduced in human colorectal cancer tissues

**Fig. 5:**
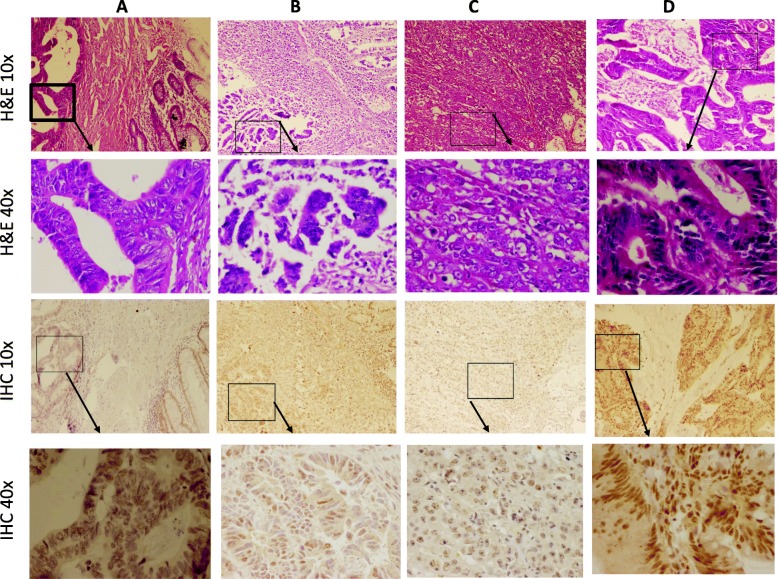
Representative images of H&E and IHC of ARID1A from four CRC tumors. **a** Negative expression; **b** Low expression; **c** Moderate expression; and **d** High expression of ARID1A

Immunohistochemistry was conducted on 18 paired normal CRC samples. Expression of ARID1A in adjacent nontumor mucosal epithelial cells was present in all patients. IHC analysis revealed that ARID1A protein expression was negative in 7 out of 18 (38.8%) tumor tissues when compared with adjacent non-malignant counterparts. Five (27.7%) tumors had low expression of ARID1A, 4 (22.2%) tumors had moderate expression of ARID1A, and 2 (11.1%) tumors had high expression of ARID1A. Thus, a total of 12 of 18 (66.6%) CRCs demonstrated negative or low ARID1A expression when compared with normal tissues. Collectively, these results demonstrate that ARID1A is frequently down regulated in CRC tumors. Typical immunostaining of high, moderate, low and negative expression of ARID1A are shown in Fig. 5.

### ARID1A expression is not associated with clinicopathological parameters and survival rates in patients

Relationships between *ARID1A* expression and clinicopathological features was analyzed in patients with colorectal cancer. Table 1 summarizes the clinicopathologic data of the 18 CRC patients with statistical results. Among the 18 CRC patients tested, six (33.33%) were MSI-L/EMAST, five (27.7%) were MSI-high and the other seven (38.8%) were MSS. Loss of ARID1A expression was more common in CRCs arising in rectum (71.42% of all ARID1A-negative tumors vs. 54.54% of all ARID1A-positive tumors) and female (42.85% of all patients with loss of ARID1A vs. 27.28% of all patients without loss of ARID1A) and in association with both negative lymph vascular invasion and TNM stage III (71.42 and 57.14% of all ARID1A-negative tumors, respectively). Overall, MSI and MSI/EMAST are less represented in the no and low ARID1A expression group while lymphatic penetration seems to be more prominent in comparison with moderate and high expression group (33% VS. 16.66% respectively; *p* = 0.457). These trends failed to reach statistical significance. Overall, there was no significant association between the loss of ARID1A expression and any clinicopathological parameters examined.

**Table 1:** Association between ARID1A expression and clinicopathologic characteristics in colorectal cancer, n (%)

Characteristics	ARID1A				*P* value
	Negative (*n* = 7)	Low (*n* = 5)	Moderate (*n* = 4)	High (*n* = 2)	
**Age at diagnosis**					0.643
≤ 50	2 (28.58%)	2 (40%)	2 (50%)	0 (0%)	
> 50	5 (71.42%)	3 (60%)	2 (50%)	2 (100%)	
**Gender**					0.534
Male	4 (57.14%)	4 (80%)	2 (50%)	2 (100%)	
Female	3 (42.85%)	1 (20%)	2 (50%)	0 (0%)	
**Tumor location**					0.104
Right Colon	2 (28.57%)	1 (20%)	2 (50%)	1 (50%)	
Left colon	0	0 (0%)	0 (0%)	1 (50%)	
Rectal	5 (71.42%)	4 (80%)	2 (50%)	0 (0%)	
**TNM stage (AJCC)**					0.178
I	1 (14.28%)	2 (40%)	2 (50%)	0 (0%)	
II	2 (28.58%)	0 (0%)	1 (25%)	2 (100%)	
III	4 (57.14%)	3 (60%)	1 (25%)	0 (0%)	
**Pathologic differentiation**					0.420
Poor	1 (14.28%)	1 (20%)	0 (0%)	1 (50%)	
Moderate	2 (28.58%)	0 (0%)	0 (0%)	0 (0%)	
Well	4 (57.14%)	4 (80%)	4 (100%)	1 (50%)	
**Tumor size (cm)**					0. 762
≤ 5	2 (28.57%)	2 (40%)	1 (25%)	0 (0%)	
> 5	5 (71.42%)	3 (60%)	3 (75%)	2 (100%)	
**MSI status**					0.808
MSI-H	1 (14.28%)	2 (40%)	1 (25%)	1 (50%)	
MSS	3 (42.85%)	2 (40%)	2 (50%)	0 (0%)	
MSI-L/EMAST	3 (42.85%)	1 (20%)	1 (25%)	1 (50%)	
**Lymphatic penetration**					0.725
Negative	5 (71.4%)	3 (60%)	3 (75%)	2 (100%)	
Positive	2 (28.6%)	2 (40%)	1 (25%)	0 (0%)	

*TNM* Tumor-node metastasis, *AJCC* American Joint Committee on Cancer, *ARID1A* AT-rich interactive domain 1A, *EMAST* elevated microsatellite alterations at selected tetranucleotide repeats, *MSI-H* microsatellite instability-high, *MSI-L* microsatellite instability-low; MSS: microsatellite stable

**Fig. 6:**
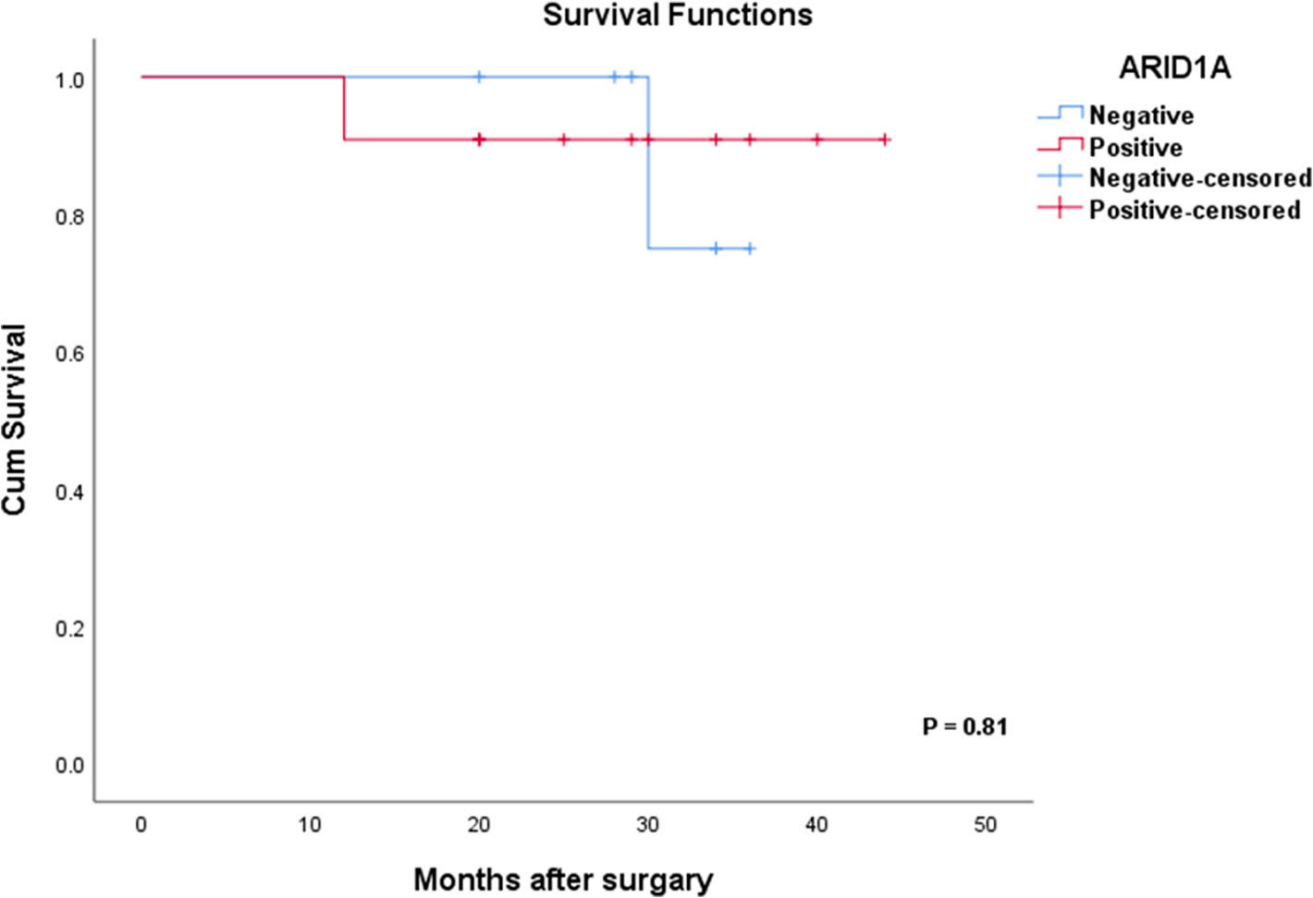
Kaplan-Meier curves. Kaplan-Meier curves with log-rank test for patients with positive ARID1A expression vs. negative ARID1A expression

In addition, we further evaluated the relationship between ARID1A expression and overall survival in colorectal cancer patients. The mean duration of follow up was 29.18 months, ranging 12–44 months, during which 2 patients died of the disease. Kaplan-Meier survival analysis showed that overall survival was worse for patients with positive ARID1A expression than the ones with negative ARID1A expression, although it was not statistically significant (Fig. 6). The Kaplan-Meier curve highlights the need for a larger number of patients to confirm the prognostic value of ARID1A expression in colorectal cancer.

Exome data analysis from 12 pairs of cancer-matched normal tissues revealed that only one patient had mutations in *ARID1A*. The patient was KRAS wild type and displayed mutations in MSH6 and PKHD1 genes (See supplemental table).

## Discussion

Loss of ARID1A expression is frequent in many types of cancers. Previous studies [21, 37] showed that almost 60% breast invasive ductal carcinoma exhibit loss of ARID1A expression and is associated with the most aggressive phenotypes of breast cancer. A study also showed that the lost expression of ARID1A was common in 30% of gastric cancer patients and was associated with poor clinical prognosis [38]. Current evidence on the importance of ARID1A shows that this gene acts as a tumor suppressor, and plays a key role in the progression of many types of cancer [13]. However, there are few studies that investigated the incidence and clinicopathologic importance of ARID1A loss in colorectal cancer and available published results are not conclusive [21, 23, 39]. For instance, Wei et al. showed that loss of ARID1A expression was associated with distant metastasis and late TNM stage of CRC; however, they did not find any association between loss of ARID1A expression and age, gender, tumor location and tumor size [21]. Whereas Chou et al. found that ARID1A loss was significantly associated with age, gender, tumor location and tumor size; however, they reported no association between ARID1A loss of expression and distant metastasis and TNM stage of CRC [23]. These contradictory results led us to investigate the incidence of ARID1A expression loss in CRC patients and analyze any association with clinicopathological parameters. Interestingly, our IHC results revealed that ARID1A was frequently reduced in CRC tissues when compared with paired normal tissues. Our results showed no ARID1A protein expression in 38.8% (7/18) cases of colorectal cancer and 66.6% had no or low expression. This suggests that ARID1A expression loss contributes to oncogenic transformation. Although we did not find any significant correlation between loss of ARID1A expression and both clinicopathological parameters and survival rates, the 66.6% incidence of negative and low ARID1A expression suggests that it is an important driver event in a large proportion of CRCs. In line with our results, Wei et al. observed that 25.8% of colorectal cancer tumors had loss of ARID1A expression and 51.2% of the tumors had low ARID1A expression compared with adjacent non-malignant tissue samples [21]. Overall, current IHC results and some previous studies [21, 23, 39, 40] suggest that ARID1A loss is not rare in colorectal cancer tumors. However, the molecular basis of this downregulation remains to be elucidated.

With regard to the clinicopathologic correlates and ARID1A expression loss, our investigation revealed no significant association between the loss of ARID1A expression and any clinicopathological parameters examined, including MSI status. Our results are in line with the study of Lee et al. [39]. These authors reported that ARID1A expression loss was not related to MSI status, TNM stage, pathologic differentiation, tumor location and tumor size. Similar findings were also reported by Rahman et al. [41], who reported no significant association between ARID1A loss and common clinicopathological parameters in uterine endometrioid carcinomas. In this context, it is also worth noting that in a study by Katagiri et al. using cervical adenocarcinomas showed high frequency of ARID1A expression loss but reported no significant association between the loss of ARID1A expression and any clinicopathological parameters. These authors speculated that one possible explanation for lack of correlation is that loss of ARID1A expression is an early event in the development of cancer. ARID1A protein might act as a “brake” by preventing excessive cellular proliferation and is essential for normal cell cycle arrest. This suggests that loss of ARID1A protein expression might not be as critical to tumor progression as to tumor initiation [42]. Notably, inconsistent reports also exist [21, 23, 40, 43]. Collectively, with respect to clinicopathologic importance of ARID1A loss, the documented results to date are controversial. These discrepancies might be attributed to differences in sample sizes, tissue- and organ-specific tumorigenesis pathways and different types of used IHC antibodies (mono vs. polyclonal).

Our study showed no significant association between overall survival and loss of ARID1A expression in CRC. However, patients with negative ARID1A expression had a tendency towards better overall survival than those with positive ARID1A expression. Our results are consistent with previous reports on CRC [23, 39, 40]. The previous studies did not find any significant association between loss of ARID1A expression and overall survival; however, they observed that overall survival was better for patients with negative ARID1A expression than those with positive ARID1A expression [23, 39, 40]. Only one previous study found a significant association between loss of ARID1A expression and overall survival in stage IV colorectal cancer, rather than in stage I-III patients. However, the small number of patients examined in the current study is not sufficient to determine the prognostic impact of ARID1A expression in CRC. Hence, further studies are required to clarify the prognostic value of ARID1A in CRC.

In order to characterize molecular mechanisms affecting ARID1A expression, we evaluated the possible role of promoter methylation in the downregulation of the ARID1A expression in various types of colorectal cell lines. A study showed that *ARID1A* promoter methylation led to decreased ARID1A expression in breast cancer [17]. However, there has been no investigation on *ARID1A* epigenetic inactivation in colorectal cancer. The present study evaluated *ARID1A* promoter methylation, and its association with the ARID1A expression levels in human CRC cell lines. *ARID1A* mRNA level was different in colorectal cancer cell lines and varied from almost nondetectable in SW48 to high expression level in HCT116 and HT-29/219. Hypermethylation was further detected in 4/6 (66%) CRC cell lines. This study showed that the SW48 cell line, displaying the *ARID1A* methylated allele only, did not express detectable *ARID1A*. In contrast, HCT116 and HT-29/219 displayed an unmethylated allele and a methylated allele, which was not detectable. We have also shown that treatment with the 5-aza dramatically restored ARIA1A expression in low *ARID1A* expressing cell lines, SW48 and SW742.

To sum up, the present study identified that promoter DNA hypermethylation strongly contributes to *ARID1A* silencing or downregulation in the CRC cell lines. However, other processes such as histone modification cannot be excluded. Indeed, ARID1A expression was not restored after 5-aza treatment in SW480 and LS180 cell lines. Zhang et al. carried out chromatin immunoprecipitation assays to evaluate *ARID1A* promoter histone modification levels in breast cancer samples and reported methylation of histone H3 at lysine 27 was linked to *ARID1A* silencing [17]. These findings suggest that different regulatory processes might play a role in ARID1A expression in different CRC lines. ARID1A is regulated by DNA methylation and repressive histone modifications and as such likely acts as a potent tumor suppressor gene in some subtypes of colorectal tumors, as it affects many genes’ expression through its role in chromatin remodeling.

As previously mentioned, a significant association between loss of ARID1A expression and MSI because of hypermethylation of the MLH1 gene promoter was reported in various cancers, including CRC [26, 40, 43]. It was speculated that ARID1A expression loss would result in aberrant epigenetic alterations by a deficient SWI/SNF complex with subsequent promoter hypermethylation of the MLH1 gene. Alternatively hypermethylation of the *ARID1A* promoter could be considered as an inactivating mechanism of ARID1A expression, and the co-occurrence of loss of ARID1A expression and MLH1 promoter hypermethylation might be the consequently a reflection of global genomic hypermethylation [26, 43]. This last hypothesis might be applicable to colorectal tumors, as we identified promoter hypermethylation as a silencing or downregulation mechanism of the *ARID1A* gene in the CRC cell lines. On a different note, lymphatic penetration was more pronounced in no/low ARID1A expression cases when compared to moderate/high expressing cases (33% vs. 16.66%). This finding suggests that lack of ARID1A expression renders the tumors more aggressive and invasive to neighboring tissues. Indeed, Kishida et al. have reported that negative ARID1A expression was correlated with early onset, lymphatic invasion, and lymph nodes metastasis and was also associated with specific mutational profile in PKHD1, RNF213, and MSH6 genes whereas KRAS mutations were more common in ARID1A-positive tumors [44].

Previous studies showed that copy number loss in *ARID1A* was the major mechanism for loss of expression in pancreatic cancer [28]. In addition, several previous studies indicated copy number loss occurs in 13–35% in breast cancers [17]. These led us to investigate whether copy number loss is also involved in the low ARID1A expression in the CRC cell lines. The comparison between *ARID1A* copy number in the CRC cell lines and normal sample revealed that CRC cell lines had no significant difference in *ARID1A* copy number and values were similar to the control sample (Fig. 3). These data suggest that the CNV is not for a player in the observed differences in ARID1A expression in the CRC cell lines.

This study has several limitations. The possible significance of *ARID1A* promoter hypermethylation in CRC requires further elucidation including complete CpG island promoter analysis that will be our goal for future studies using previous protocols [45]. In addition, the correlation between the ARID1A expression and promoter methylation requires to be assessed in patients with CRC and in a greater number of CRC cell lines. We are aware of other epigenetic mechanisms of silencing such as aberrant methylation of an upstream regulatory gene, dysregulation of miRNA expression, mutations and deletion that can possibly play a role in ARID1A expression in CRC.

In the case of our finding that loss of ARID1A expression was not correlated to survival and clinicopathological parameters, the limited number of patients might have limited our analysis. Further investigations with a larger number of patients are warranted to establish clinical and pathological impact of ARID1A expression loss in CRC.

## Conclusion

We found a high incidence of ARID1A expression loss in CRC that suggests ARID1A as a tumor suppressor. There was no significant association between the loss of ARID1A expression and any of the clinicopathological parameters examined. Here we identified aberrant methylation of *ARID1A* gene as an important regulator of ARID1A expression in CRC cell lines and clinical specimens. The clinical significance of loss of *ARID1A* in CRC remains a goal for future studies.

## Supplementary information

**Additional file 1.**

## Data Availability

All data generated or analyzed during this study are included in this published article.

## Abbreviations

ARID1A: AT-rich interactive domain-containing protein 1A
CRC: colorectal cancer
MMR: Mismatch repair
EMAST: Elevated microsatellite alterations at selected tetranucleotide repeats
MSI: Microsatellite Instability
CNV: Copy number variation
qPCR: Real-time quantitative PCR
